# The Effect of Diverse Linguistic Experience on Second Language–Accented English Sentence Recognition Among Monolingual and Spanish–English Bilingual Children and Adults

**DOI:** 10.1044/2025_JSLHR-24-00695

**Published:** 2025-12-19

**Authors:** Tiana M. Cowan, Emily Buss, Lori J. Leibold, Anne J. Olmstead

**Affiliations:** a Center for Childhood Deafness, Language and Learning, Boys Town National Research Hospital, Omaha, NE; b Department of Otolaryngology—Head and Neck Surgery, University of North Carolina, Chapel Hill; c Center for Hearing Research, Boys Town National Research Hospital, Omaha, NE; d Department of Communication Sciences and Disorders, Pennsylvania State University, University Park

## Abstract

**Purpose:**

This project evaluates whether diverse linguistic experience influences second language (L2)–accented English sentence recognition in Spanish–English bilingual and English monolingual school-age children and adults.

**Method:**

Four groups of participants completed the study: 22 Spanish–English bilingual school-age children, 21 English monolingual school-age children, 19 Spanish–English bilingual adults, and 19 English monolingual adults. Participants completed English sentence recognition and English language testing. Sentence recognition was assessed in speech-shaped noise at ascending target-to-masker ratios in three accent conditions: Korean (unfamiliar to all), Midland (familiar to all), and Spanish (familiar to the bilingual group). Speech recognition thresholds associated with 50% correct performance (SRT50) and asymptotic performance were extracted from psychometric function fits to behavioral data.

**Results:**

Adults had lower (better) SRT50s in the Spanish and Korean accent conditions compared to children. Estimates of recognition in quiet were better for bilingual children tested in the Spanish and Korean accent conditions compared to monolingual children. The effect size was larger in the Spanish accent condition. There were no language group differences in performance among adults.

**Conclusions:**

For children, prior experience with a specific L2 accent offers significant benefit in accurately recognizing sentences spoken by talkers of that accent. This effect is not observed in adults, potentially reflecting differences in language experience between Spanish–English bilingual child and adult participants. Although greater diversity in linguistic input is also positively associated with recognition of unfamiliar accented speech in children, its effect is smaller than that of accent-specific experience.

**Supplemental Material:**

https://doi.org/10.23641/asha.30827552

Children frequently encounter linguistic diversity, including different languages and second language (L2) accents, in their everyday interactions at home, in school, and throughout their communities (U.S. Census Bureau, 2019). However, few studies have examined how children's linguistic experiences influence their ability to recognize unfamiliar accented speech. While adults efficiently adapt to speech produced with an unfamiliar accent, children are less accurate at recognizing it, in part due to their more limited linguistic experience (e.g., Bent, 2014). Recognition of unfamiliar L2-accented speech develops gradually throughout childhood and adolescence; even by 14–15 years of age, children still do not achieve adultlike recognition in the presence of background noise (Bent, 2018). Most research on school-age children's recognition of L2-accented speech has evaluated English monolinguals, primarily focusing on the roles of vocabulary size and phonological awareness rather than the influence of linguistic experience (Bent, 2014, 2018; Bent & Holt, 2017; see also Mulak et al., 2013). The present study evaluates whether diversity in linguistic experience predicts L2-accented speech recognition in Spanish–English bilingual and English monolingual children and also compares children to adults within each language group to assess age group–related differences in performance.

## Theoretical Frameworks of Speech Recognition in Children and Adults

Encountering talkers with unfamiliar accents introduces linguistic variation that listeners must contend with when mapping speech input to phonemes and phonological features (see Bent, 2018, for a review). This mapping process integrates bottom-up processing of acoustic–phonetic cues with top-down processing of linguistic information (e.g., Burgess & Chiarello, 1996; Marslen-Wilson & Welsh, 1978). Mismatches reduce speech recognition accuracy and processing speed (Creel, 2012; Munro & Derwing, 1995). Despite this initial processing cost associated with recognizing unfamiliar speech, experience with a specific talker or speech pattern facilitates more accurate recognition. For example, L2 accents reflect linguistic patterns across multiple levels of a talker's languages, including speech sound inventories, prosodic patterns, and suprasegmental features, and are shaped by the bidirectional influence between the talker's languages as well as by their language use over time (e.g., Flege et al., 2021). This systematicity helps listeners extract regularities from the speech signal and apply them to recognize speech produced with an L2 accent more accurately (e.g., Norris et al., 2003).

Vocabulary size is hypothesized to predict L2-accented word recognition, because it drives phonological refinement (Metsala, 1999), supporting the mapping of unfamiliar pronunciations to known words (Bent & Atagi, 2015; Brosseau-Lapré & Kim, 2020; Creel et al., 2016). The lexical restructuring hypothesis posits that children's phonological representations start as whole word units and become more detailed with vocabulary growth, evolving from segments (e.g., syllables) to finer phonetic details (Walley, 1993). According to this hypothesis, as a child's vocabulary expands, they encounter words with similar features, necessitating representations with more precise phonetic detail for identification. Experimental evidence supports the lexical restructuring hypothesis in monolingual development, showing that vocabulary size predicts performance in word recognition (e.g., Ventura et al., 2007) and phonological awareness skills like word segmentation and blending syllables and phonemes into words (Ainsworth et al., 2016; Krenca et al., 2023; Metsala, 1999). Preliminary evidence suggests that the lexical restructuring hypothesis also applies to bilingual development, with vocabulary size predicting phonological awareness in each language (Scarpino et al., 2011). Additionally, studies on L2-accented speech perception in both monolingual (Bent & Atagi, 2017) and bilingual (Hu, 2021) children show positive correlations between target language vocabulary size, nonword repetition scores, and speech recognition performance. However, other factors that could influence phonological refinement and L2-accented speech recognition beyond vocabulary, such as diverse linguistic experience, have not been as thoroughly evaluated in school-age children.

Accounts differ with respect to the roles of exposure to a specific accent and diverse linguistic experience on the recognition of talkers with L2-accented speech (see Bent & Baese-Berk, 2021, Johnson et al., 2022, and Quam & Creel, 2021, for reviews). Some accounts emphasize the benefit of exposure to a specific L2 accent, suggesting that listeners modify their phonological representations to incorporate the acoustic–phonetic properties of that accent, leading to improved recognition (e.g., Norris et al., 2003). In contrast, other accounts propose that multiple processes underlie speech recognition and adaptation, allowing listeners to generalize to unfamiliar speech patterns without direct exposure (Bradlow & Bent, 2008; Clarke & Garrett, 2005; Levi, 2024). For instance, Cowan and Olmstead (2023) suggest that diverse linguistic experience helps listeners develop flexible strategies that enhance their ability to recognize unfamiliar L2-accented speech (see also Potter & Saffran, 2017). Findings from studies involving adults support this hypothesis, showing that meta-linguistic skills, such as sensitivity to indexical information, predict greater accuracy in recognizing speech produced with an unfamiliar accent (Atagi & Bent, 2013). Another possibility is that diverse linguistic experience enables listeners to extract a broader range of phonetic features, facilitating generalization to unfamiliar L2-accented speech patterns more effectively than exposure to a single language, dialect, or accent. For example, many L2 accents in English exhibit reduced unstressed vowel reduction and a slower speech rate compared to native English speech, which may facilitate accent-independent generalization to untrained or unfamiliar L2-accented speech (Baese-Berk et al., 2013; see also Porretta et al., 2016).

The present study extends prior work to school-age children by assessing English sentence-in-noise recognition among Spanish–English bilingual and English monolingual children. The results will allow us to explore whether differences in linguistic experience influence how children recognize unfamiliar L2-accented speech. Spanish–English bilingual children living in the Midwest hear Spanish at home and are likely exposed to a broad range of linguistic input, including Spanish-accented English and Midland English, on a regular basis. In contrast, the English monolingual children in this study are only exposed to Midland English at home, making their overall experience less diverse compared to the bilingual group. Although monolingual children may have some experience with Spanish or Spanish-accented English in community settings, this input is less frequent and likely less familiar. Since both groups are exposed to Midland English, this accent serves as the reference accent condition in the present study. Leveraging these group-level differences, this study examines whether increased diversity in linguistic experience at home and in the community supports greater flexibility in recognizing unfamiliar L2-accented speech.

## Speech Recognition in Noise

Most natural listening environments contain multiple sound sources, and the success of speech communication in these environments depends on the ability to recognize speech in noise. This noise often overlaps with the spectral energy of target speech and disrupts peripheral encoding of that signal, reducing audibility for the listener (Brungart et al., 2001; Garcia Lecumberri et al., 2010). Whereas young children tend to require a more favorable signal-to-noise ratio than adults to perceive speech in the presence of noise, performance is adultlike by 10 years of age for most speech-in-noise tasks (Buss et al., 2012; Eisenberg et al., 2000). Notably, much of this research used target speech that matches the dominant dialect of the community and tested English monolingual children. The developmental trajectory for recognizing unfamiliar-accented speech in noise remains underexplored. This is particularly important to evaluate, as the effects of competing noise may be more disruptive for recognizing speech based on unfamiliar phonetic cues than for familiar ones that are encountered regularly.

### Factors That Influence Speech Recognition in Noise for Bilinguals and Monolinguals

Recognizing speech in noise requires listeners to integrate degraded phonetic cues, a process influenced by experience (Shi & Canizales, 2013). The efficiency of phonetic cue integration in noise is influenced by how frequently a listener encounters the target speech sounds and words in their everyday input (Schmidtke, 2016). Bilingual individuals tend to be more susceptible to noise in their nondominant language compared to their monolingual peers, in part due to limited exposure to the target language (Desjardins et al., 2019; Mayo et al., 1997; Regalado et al., 2019; Rogers et al., 2006; Tabri et al., 2010). Individual differences in linguistic factors associated with differences in frequency of exposure, such as language history, predict resilience to masking for bilingual listeners (see Cowan et al., 2022, for a review). For bilingual children, linguistic factors, such as age of exposure, cumulative exposure, and target language competency predict resilience to noise. (Miller et al., 2019; Reetzke et al., 2016). For example, Miller et al. (2019) assessed masked English speech recognition in Spanish–English bilingual and English monolingual children and found that both younger and older groups of bilingual children displayed similar speech recognition capabilities in noise compared to their monolingual peers when English receptive vocabulary scores and maternal education were controlled (see also Strydom et al., 2022). These findings suggest that, when bilingual children have sufficient exposure to the target language, they attain speech-in-noise recognition comparable to their monolingual peers.

## L2-Accented Speech Recognition in Bilingual Children

Few studies have examined how bilingual school-age children perceive L2-accented speech, and their methodological limitations have made it challenging to draw meaningful conclusions about how diverse linguistic input impacts speech recognition. Much of the existing literature has focused on how monolingual children respond to experimental training paradigms rather than evaluating the effects of long-term experience with linguistic variation (e.g., Levi, 2015). Another limitation of the existing literature includes confounds in group comparisons between monolingual and bilingual children (e.g., socioeconomic status, target language vocabulary size). For instance, Levy et al. (2019) tested the hypothesis that diverse linguistic input could generalize to talkers with unfamiliar dialects and L2 accents. Specifically, they evaluated whether bilingual experience increases the number of phonemic contrasts in children's input, thereby enhancing sensitivity to speech sound productions and improving recognition of unfamiliar accented speech (e.g., Fox et al., 1995). Levy et al. recruited German monolingual and bilingual school-age children with varied language backgrounds and assessed familiarity with dialects of German and L2-accented German. Monolingual children had higher German vocabulary scores than the bilingual group and reported greater exposure to different German dialects, whereas bilingual children reported greater exposure to L2 accents. The stimuli were meaningless sentences recorded by talkers who either spoke the Palatinate German dialect or Korean-accented German. Sentence recognition was measured in quiet. Results indicated that children with greater exposure to different dialects were more accurate at perceiving an unfamiliar dialect. However, that benefit was not observed for L2 accent exposure. Levy et al. interpret this finding to suggest that phonetic representations are formed per language system and reflect phonetic features from that input; they hypothesized that exposure to variability within a language generalizes to talkers with unfamiliar dialects, but exposure to L2-accented speech does not generalize to new L2 accents. However, language group differences in German vocabulary size complicate this interpretation (Levy et al., 2019).

While adult bilinguals are sensitive to phonological contrasts in unfamiliar accents when those contrasts exist in one of the languages that they speak (e.g., Cooper & Bradlow, 2018), this effect is not evident in the limited data collected on children. Studies comparing bilingual and monolingual L2-accented speech recognition have reported no significant difference in behavioral performance related to experience with specific (McDonald et al., 2018) or diverse linguistic input (Levi, 2024). McDonald et al. (2018) recruited three groups of 5- and 6-year-old children to compare how language status predicted the comprehension of Spanish-accented English, a speech pattern familiar to two of the three groups. They compared how English monolingual, “simultaneous” Spanish–English bilingual, and “early” Spanish–English bilingual groups judged the semantic accuracy of meaningful and nonsense English sentences produced with a Spanish accent. All stimuli were presented in quiet. Results indicated that there were no group differences for meaningful sentences, but that simultaneous bilinguals were more accurate compared to monolingual and early bilingual groups in the nonsense sentence condition. The authors interpreted these findings to suggest that prior experience with speech with similar phonetic patterns (i.e., Spanish-accented English) may not confer a measurable advantage in typical everyday listening contexts, where semantic context supports recognition. In contrast, when semantic support is reduced, as in the nonsense sentence condition, familiarity appears to facilitate recognition.

There are two recent studies that evaluated how experience with diverse linguistic input affects recognition of L2 accented speech, while controlling for proficiency in the test language (Hu, 2021; Levi, 2024). Levi (2024) compared school-age children (ages 6.7–11.8 years) who only heard English at home with those who heard English and at least one other language. Neither group had prior exposure to speech produced with a German-accented English, and the groups did not differ significantly on standardized measures of English receptive vocabulary. Findings indicated that German-accented word recognition did not significantly differ between groups. However, working memory only predicted performance for the English-only group and not the group exposed to multiple languages. Levi interprets this finding to suggest that diverse linguistic experience may refine phonological representations in ways that reduce cognitive load during the perception of speech produced with an unfamiliar accent. In another recent study, Hu (2021) explored the impact of short-term exposure on adaptation to speech produced in Indian English (IE). In this study, Mandarin–English bilingual children listened to a story read by either a Mandarin-accented talker or IE talkers, and completed a word recognition task before and after exposure. Children exposed to IE speech showed improved recognition of trained words, though this improvement did not generalize to untrained words. Improvement in performance from pre- to posttest was predicted by English phonological short-term memory, phonemic awareness, and receptive vocabulary scores, consistent with previous research showing that precise phonological representations in the target language predict better recognition of unfamiliar dialects or accents (Bent & Atagi, 2017; Levy et al., 2019).

Together, these studies highlight the need to better understand how diverse linguistic experience influences L2-accented speech recognition in children. While some findings suggest that diverse linguistic experience supports the perception of speech produced with an L2 accent, others indicate that such benefits may be limited to a subset of conditions. Additionally, differences in target language receptive vocabulary size between groups have made it difficult to interpret the effects of experience in some prior research. To address these gaps, the present study tests the hypothesis that diverse linguistic experience generalizes to unfamiliar L2-accented speech. Specifically, we examine whether such experience facilitates recognition of Spanish- and Korean-accented English sentences in noise among bilingual and monolingual children with comparable English vocabulary knowledge.

## The Present Study

The current study evaluated how diverse linguistic experience predicts the recognition of English sentences produced by talkers with familiar and unfamiliar L2 accents during childhood. To address this question, sentence recognition in speech-shaped noise (SSN) was compared across three distinct accent conditions where the familiarity with the phonetic features of those accents was assumed to vary between bilingual and monolingual participants. Specifically, the present experiment measured English sentence recognition in SSN for three target speech accent conditions: Midland (familiar to all participants), Spanish (assumed familiar to bilingual participants), and Korean (assumed unfamiliar to most participants). The Midland accent was selected because it is the dialect most spoken in Nebraska, so it is likely familiar to all participants (Schneider, 1996). In the Midland accent condition, we expected speech recognition thresholds (SRTs) associated with 50% correct performance (SRT50) to be similar for children and adults, as the children in this study are between 7 and 15 years old, and both language groups demonstrate high English language proficiency and regular use. This expectation is based on prior findings that performance tends to converge on adultlike scores by 10 years of age when listeners are proficient in the target language and the speech is produced in a familiar English dialect (see Leibold & Buss, 2019, for a review). Spanish-accented English was selected based on the assumption that Spanish–English bilingual children and adults would have experience with both Spanish and Spanish-accented English. Korean-accented English was selected due to its linguistic distance to English (as outlined by Bradlow et al., 2010), coupled with the assumption that a Korean accent would be unfamiliar to most child participants in Nebraska, irrespective of their language status given the low proportion of Korean speakers in the population (Nebraska Department of Health and Human Services, 2021). As of 2023, approximately 11% of residents of Nebraska reported speaking Spanish at home, while fewer than 0.001% (*N* = 1,357) reported speaking Korean at home (Migration Policy Institute, 2023). If bilingual children outperform monolingual peers in both L2 accent conditions, this would support the idea that diverse linguistic experience generalizes to unfamiliar speech patterns. However, if better performance is limited to Spanish-accented English, that would suggest that the benefit relies on familiarity with features of a specific accent. A secondary purpose of this study was to compare bilingual and monolingual child groups to their respective adult reference groups to assess differences in performance across age groups in L2- and L1-accented conditions, both in quiet and in noise.

## Method

All study procedures were approved by the institutional review board at Boys Town National Research Hospital (IRB Protocol #23–21-XP). Adult participants provided written informed consent prior to participation. For child participants, written informed consent was obtained from a parent or legal guardian, and verbal or written assent was obtained from the child, as appropriate for their age.

### Materials

We used the Bilingual Input–Output Survey (BIOS; Peña et al., 2018) to create a linguistic profile for bilingual child participants and the Bilingual Language Profile (BLP; Birdsong et al., 2012) to do the same for bilingual adults.

#### BIOS

Caregivers completed the BIOS (Peña et al., 2018) to report Spanish and English input and output data for bilingual children. The BIOS asks caregivers to report this language input and output data historically, such as in preschool, as well as currently, so that it provides both data about overall language exposure and current levels of input and output. The BIOS takes caregivers approximately 10 to 15 min to complete. Caregivers could elect to complete the survey in either Spanish or English.

#### BLP

Bilingual adults completed the BLP (Birdsong et al., 2012) questionnaire, which characterizes respondents' language history, proficiency, use, and cultural attitudes and identity in four modules each yielding a module score, which range from 0 to 60. The four module scores are converted to the same scale and then are used to calculate Total Language Dominance scores, which estimate the degree of language dominance from −218 to 218. In this study, negative values indicate Spanish dominance, positive values indicate English dominance, and values near zero indicate “balanced” language dominance. The BLP takes approximately 10 to 15 min to complete. Participants could elect to complete the questionnaire in either Spanish or English.

#### English Language Tests

English language tests assessed English language skills in receptive vocabulary and phonological processing. Raw scores were recorded for all participants on these standardized language tests since the normative samples did not include Spanish–English bilinguals.

The Comprehensive Test of Phonological Processing–Second Edition (CTOPP-2; Wagner et al., 2013) assesses phonological skills that are predictive of reading outcomes in children and is normed for English monolinguals ages 4–24 years. There are six core tests in the CTOPP-2, and three were administered in this study: blending, elision, and nonword repetition. Blending asks participants to take small parts of words and combine them into a full word. Elision asks participants to remove small parts of words from whole words. Together, these two subtests assess English phonological awareness skills. Nonword repetition asks participants to imitate words with a lexical frequency of zero that are based on the speech sound rules of American English. Performance on the nonword repetition subtest assesses phonological short-term memory. These subtests were administered according to standardized procedures by trained research staff.

The Peabody Picture Vocabulary Test–Fourth Edition (PPVT-4, Dunn & Dunn, 2007) assesses English receptive vocabulary and is normed for English monolinguals ages 2.5–90+ years. The PPVT-4 assesses single-word receptive vocabulary in English by asking people to identify a picture that corresponds to a presented word from a field of four color illustrations (i.e., in a four-alternative forced-choice task format). The PPVT-4 was administered according to standardized procedures. The PPVT-4 is not considered a valid or reliable measure of conceptual vocabulary in bilingual children (e.g., Peña et al., 2015); however, it is commonly used in masked speech recognition research to estimate target-language vocabulary size (e.g., Leibold et al., 2024; Miller et al., 2019). In this study, the raw scores on the PPVT-4 are used for this purpose, as target language vocabulary size is a significant predictor of speech recognition in noise (Leibold et al., 2024; McCreery et al., 2020; Miller et al., 2019; Walker et al., 2019). A likely reason for this relationship is that the PPVT-4 score reflects both chronological age and cumulative English exposure.

#### Nonverbal Intelligence Testing

The Universal Nonverbal Intelligence Test–Second Edition (UNIT-2, Bracken & McCallum, 2016) assesses nonverbal intelligence and is normed for ages 5–22 years. The Analogic Reasoning and Symbolic Memory subtests were administered to describe participants' nonverbal intelligence. Both raw scores and standard scores were recorded for child participants since the manual says that the test is appropriate to use with children regardless of language background (Bracken & McCallum, 2016).

#### Sentence Stimuli

Lists 1, 2, and 3 from the Bamford–Kowal–Bench (BKB; Bench et al., 1979) sentences were used to evaluate masked speech recognition across three accent conditions (Midland, Spanish, and Korean). These accents differ in prosodic and phonetic structure in ways that may influence perception. In this study, the Spanish-accented talker (from Colombia) and the Korean-accented talker produced speech with features described in the literature, including differences in vowel production, stress placement, and consonant realization (e.g., Garrido-Pozu, 2023; Park et al., 2023). According to Labov et al. (2008), the Midland dialect spans a broad geographic region, including parts of Ohio, Indiana, Illinois, Missouri, Kansas, Iowa, Nebraska, the Dakotas, Minnesota, Wisconsin, and Michigan. A distinguishing phonological feature is the low back vowel merger, in which /ɔ/ and /ɑ/ are not contrastive. Labov et al. (2008) also note that the Midland dialect shares considerable phonological overlap with other American English dialects, making it a practical reference in this study.

The BKB sentences are semantically and syntactically simple, making them appropriate for child participants. Each sentence contains keywords that are used to assess sentence recognition accuracy. An example sentence is The CLOWN had a FUNNY FACE (keywords capitalized). After each sentence was presented, participants were asked to repeat what they heard. A keyword was marked as correct if the participant produced the word during repetition.

In the present study, sentence lists were fixed for each accent condition and talker so that all participants were exposed to the same stimuli within each condition. Specifically, all participants heard List 1 in a Spanish accent, List 2 in a Midland accent, and List 3 in a Korean accent. Within each condition, sentences were presented in a randomized order. Recordings were retrieved from the Hoosier Database of Native and Nonnative Speech for Children (Atagi & Bent, 2013; Bent, 2014), ensuring open accessibility and supporting reproducibility of the experiment. This corpus contains scripted word and sentence stimuli recorded by talkers across several language backgrounds, with four talkers (two males, two females) recorded per represented language background. Recordings were sampled at 22050 Hz. The three male talkers in this experiment are referred to in the corpus as E1 (for L1 English), S2 (for L1 Spanish), and K7 (for L1 Korean). Previous data indicate that English-speaking monolingual adults were able to correctly recognize 95% of sentences produced by talker K7 and 93% of sentences produced by talker S2 (Atagi & Bent, 2013; Bieber & Gordon-Salant, 2022). The Midland English talker had a mean sentence duration of 1.43 s (*SD* = 0.18). The L2-accented talkers spoke more slowly than the Midland English talker, with the Spanish-accented talker having a mean sentence duration of 2.01 s (*SD* = 0.23), and the Korean-accented talker having a mean duration of 1.98 s (*SD* = 0.22). A limitation of this study is that we do not have detailed demographic information about each talker within this corpus beyond their language background, country of origin, and biological sex.

Each sentence was root-mean-square normalized in MATLAB (MathWorks Inc., 2022) and spectrally shaped using a custom finite-duration impulse response filter to match the long-term average speech spectrum (LTASS) computed across all three talkers used in this study. A single SSN masker was then generated by filtering a 10-s Gaussian noise sample to match that overall LTASS. The same noise was used in all three accent conditions.

### Participants

Four groups participated: children (ages 7–15 years) and adults (ages 19–46 years)^1^ grouped by their language status, either Spanish–English bilingual or English monolingual (henceforth referred to as language groups). Spanish–English bilingual participants reported conversational fluency in both languages, and monolingual participants reported only having conversational fluency in English. All participants passed a hearing screening, with air-conduction thresholds ≤ 20 dB HL at octave frequencies from 0.25 to 8 kHz, bilaterally (American National Standards Institute, 2021). All participants resided in Nebraska at the time of testing. Caregivers reported that children had no history of neurological injury, language disorder, or learning disability. Caregivers also reported that children had no otitis media within the last 6 months. Adults self-reported no such history either. All participants' L2 accent exposure was assessed experimentally, with no significant differences between language groups. However, adults reported greater familiarity with L2 accents than children. Full methodological details and statistical results are available in the Supplemental Material S1.

#### Bilingual Children

Twenty-five Spanish–English bilingual children were recruited. Three were excluded from analysis: two for failing the hearing screen and one due to receiving speech-language intervention services. Twenty-two bilingual children (male = 9) participated in the present study. Their mean age was 10.9 years (*SD* = 2.2, range: 7–15 years). Among these bilingual children, 36.4% (*n* = 8) identified as White, 9.1% (*n* = 2) as Native American or Alaska Native, and 54.5% (*n* = 12) did not report race. Most participants (81.8%, *n* = 18) identified as Hispanic or Latino, while 18.2% (*n* = 4) did not report ethnicity. Caregivers reported that 21 (95%) child participants lived in homes with adults who were originally from Spanish-speaking countries. Eighteen caregivers (86%) self-reported speaking both Spanish and English fluently and three (14%) self-reported speaking only Spanish fluently.

Caregivers also provided information about bilingual child language history and use. Caregivers reported that bilingual children were exposed to Spanish from birth and to English by 5 years (*M* = 2.1 years, *SD* = 2.2 years). Paired *t* tests were used to compare mean values on English and Spanish input and output values from the BIOS; results indicated that, in general, bilingual children heard more English (*M* = 61%, *SD* = 23%) than Spanish (*M* = 38%, *SD* = 23%), *t*(21) = 2.12, *p* = .0454, and spoke more English (*M* = 67%, *SD* = 22%) than Spanish (*M* = 33%, *SD* = 22%), *t*(21) = 2.60, *p* = .002.

#### Monolingual Children

Twenty-four English monolingual children were recruited for this study. Three were excluded from analysis: one for a recent episode of otitis media, one due to receiving speech-language intervention services, and one who requested to discontinue speech recognition testing. Twenty-one monolingual children (male = 11) were included in the analysis. Their mean age was 10.7 years (*SD* = 2.0, range: 7–15 years), which was not significantly different from the bilingual group (*p* = .693). Of these monolingual children, 71.4% (*n* = 15) identified as White, 4.8% (*n* = 1) identified as Black or African American, and 4.8% (*n* = 1) identified as multiracial; 19.0% (*n* = 4) did not report race. Most children, 76.2%, (*n* = 16) were non-Hispanic or Latino, 4.8% (*n* = 1) identified as Hispanic or Latino, and 19.0% (*n* = 4) did not report ethnicity.

#### Bilingual Adults

Twenty-one Spanish–English bilingual adults were recruited. One was excluded due to a self-reported recent episode of otitis media, and one was excluded from analysis due to incomplete speech recognition data. Nineteen bilingual adults participated in this study (male = 5, *M* = 27 years, *SD* = 7, range: 19–46 years). Of these bilingual adults, 47.4% (*n* = 9) identified as White, 10.5% (*n* = 2) as American Indian or Alaska Native, and 42.1% (*n* = 8) did not report their race. All participants in this group identified as Hispanic or Latino. On the BLP, bilingual adults self-reported exposure to Spanish from birth and to English by 5 years (*M* = 2.6 years, *SD* = 1.5). Participants also provided self-rated language proficiency scores on the BLP, with scores ranging from 0 to 60, where higher scores indicate greater proficiency. Bilingual adults had a mean of 52 for English (*SD* = 4.7, range: 41–54) and a mean of 41 for Spanish (*SD* = 12.6, range: 7–54). Although participants rated their English proficiency higher on average than their Spanish proficiency, this difference was not statistically significant, *p* = .995. The mean Total Language Dominance score was 29 (*SD* = 45, range: −72 to 98, with positive values indicating English dominance and negative values indicating Spanish dominance), which was significantly different from zero, *t*(28) = 2.83, *p* = .011, indicating that bilingual adult participants tended to be English dominant to some degree.

#### Monolingual Adults

Twenty English monolingual adults were recruited for this study. One was excluded from analysis due to a self-reported communication-related disability. Nineteen monolingual adults participated (male = 9, *M* = 26 years, range: 19–44 years). There was no significant age difference between the monolingual and bilingual adult groups (*p* = .673). Among the monolingual adults, 89.5% (*n* = 17) identified as White, 5.3% (*n* = 1) identified as Black or African American, and 5.3% (*n* = 1) identified as American Indian or Alaska Native. Most participants, 94.7%, (*n* = 18) identified as non-Hispanic or Latino, with 5.3% (*n* = 1) identifying as Hispanic or Latino.

### Procedures

A schematic illustration of the procedures, including the randomization and blocking of tasks for all participant groups, is provided in Supplemental Material S1. All testing was completed in a single session lasting approximately 2.5–3 hr, with breaks offered as appropriate.

#### Language Testing

Participants from all four groups completed English language and nonverbal intelligence testing. The PPVT-4, CTOPP-2, and UNIT-2 were administered in a random order per participant. Language and cognitive testing was completed as a block and would occur either before or after speech recognition testing, with the order being randomized by participant. Trained research staff completed all testing.

#### Speech Recognition Testing

The presentation of sentences by accent condition was controlled using custom MATLAB software (MathWorks Inc., 2022). Sentences were mixed with noise, routed to a GSI 61 audiometer, and presented diotically over Telephonics TDH-50P supra-aural headphones, which have a frequency response that is flat to within ±3 dB up to 6 kHz and then declines by approximately 5 dB between 6 and 8 kHz. Participants were instructed to repeat back target sentences and ignore the background noise. An experimenter sat outside the sound booth, monitored participant responses over headphones, and performed keyword scoring. Each accent condition contained one list of 16 sentences. The SSN played continuously at 70 dB SPL. SRTs were measured using an ascending talker-to-masker ratio (TMR) procedure in which sentences are presented at multiple TMRs (Buss et al., 2015). In this procedure, each sentence was repeated at increasing TMRs until all keywords were recognized correctly. Once the participant correctly recognized all keywords in a sentence, 100% correct recognition was assumed for higher TMRs. For adults, TMR values were − 10, −7.5, −5, −2.5, 0, 2.5, 5, 7.5, and 10 dB. For children, values spanned −5 to 10 dB in steps of 2.5 dB. If the participant did not report the sentence correctly at the maximum TMR (10 dB TMR for both age groups), then it was presented in quiet. This procedure allowed for the estimation of asymptotic performance. The order of accent conditions was randomized by participant.

The data for each participant and condition were fitted with a logit function to characterize percent keyword recognition by TMR. The function is defined by the equation γ = Γ /(1 + exp[−(*x*−SRT50)/β]), where γ is the predicted proportion of correct responses, *x* is the level in dB TMR, SRT50 represents the midpoint of the function, β denotes the slope of the function at the inflection point, and Γ indicates asymptotic performance. For the purpose of fitting this function, performance in quiet was represented with a TMR of 20 dB.

### Analysis

The dependent variables of interest were the SRT50 and asymptotic performance for each accent condition. All parameters were derived from psychometric function fits to the behavioral data. Linear mixed models evaluated how fixed effects and random effects predicted performance related to SRTs. Beta regression models assessed how fixed and random effects predicted asymptotic performance, as this method is robust to heteroskedasticity and skewness, which are common in this type of proportion data (Cribari-Neto & Zeileis, 2010). Because the upper asymptote values were bounded and skewed toward 1, those data were transformed prior to analysis using the equation γ * (*n* − 1) + 0.5) / *n*. This transformation adjusted the values to fall within the boundaries of 0 and 1 while maintaining their relative proportions, in line with the recommendations of Smithson and Verkuilen (2006)*.* Full models including adult and child data included the three-way interaction of language group (bilingual, monolingual), age group (child, adult), and accent condition (Midland, Spanish, and Korean). To account for the repeated measures design, all models contained random intercepts by participant. Models including only child data were similar in structure, but included additional predictors of interest, such as chronological age in years and raw receptive English vocabulary scores. Model assumptions were assessed using typical procedures, such as residual assessment, model fit assessment, and review of variance components estimates.

Pairwise comparisons evaluated the mean data of language or age groups following the identification of significant interactions involving three or more categorical variables (e.g., age group, language group, accent condition; Searle et al., 1980). The Tukey method accounted for multiple comparisons. Analyses were conducted in RStudio (RStudio Team, 2022) using the packages lme4 (Bates et al., 2015), glmmTMB (Brooks et al., 2017), and emmeans (Lenth & Piaskowski, 2025), as well as MATLAB (MathWorks Inc., 2022).

## Results

Descriptive statistics for all English language measures by group are presented in Table 1. Language group differences in performance on language measures were assessed by two-sample *t* tests or Welch's two-sample *t* tests, as appropriate (results reported in Table 1). There were no significant differences in nonverbal intelligence scores between bilingual and monolingual groups. Among child groups, there was no significant difference in mean English receptive vocabulary scores between bilingual and monolingual participants. Monolingual children had significantly higher nonword repetition raw scores (*M* = 18.05) than bilingual children (*M* = 15.77), *t*(41) = −2.20, *p* = .034. Overall, the child language groups did not differ on key cognitive and linguistic factors, facilitating the examination of the influence of experience on L2-accented speech perception.

**Table 1.** Descriptive statistics showing the mean, standard deviation, and range of raw scores on English language and nonverbal intelligence testing by group and comparisons of mean scores by language group for child and adult participants.

Measure	Age group	*t*	*df*	*p*	*M* (*SD*) Range	
					Bilingual group	Monolingual group
Analogic reasoning	Children	−0.61	41	.545	27.95 (10.6) 15–48	29.81 (9.2) 18–47
	Adults	−1.09	35	.285	41.73 (7.8) 24–52	44.33 (6.7) 23–51
Nonsymbolic reasoning	Children	−0.70	39	.490	18.23 (8.7) 7–36	19.86 (6.5) 12–38
	Adults	−1.41	31	.170	32.32 (8.3) 14–43	35.50 (5.2) 21–41
Receptive vocabulary	Children	−1.72	41	.092	153.59 (26.6) 118–214	167.90 (28.0) 115–212
	Adults	−2.25	35	.031*	204.47 (9.2) 185–221	210.68 (7.8) 195–224
Nonword repetition	Children	−2.20	41	.034*	15.77 (3.2) 7–23	18.05 (3.6) 11–27
	Adults	−1.84	35	.074	17.37 (2.5) 13–22	19.00 (3.0) 14–25
Blending	Children	−1.93	41	.060	22.68 (3.6) 16–31	24.71 (3.3) 19–30
	Adults	−2.44	31	.021*	25.15 (4.5) 15–31	28.16 (2.9) 22–32
Elision	Children	−1.41	41	.166	24.82 (6.9) 13–33	27.33 (4.5) 15–32
	Adults	−0.40	33	.689	30.32 (3.7) 18–34	30.74 (2.7) 26–34

*Note.* This table presents comparisons of language measure and nonverbal intelligence raw scores between bilingual and monolingual language groups. Language group comparisons were made by age group. Raw scores are shown for the following measures: two subtests from the Universal Nonverbal Intelligence Test–Second Edition: analogic reasoning, nonsymbolic reasoning; the Peabody Picture Vocabulary Test; three subtests from the Comprehensive Test of Phonological Processing–Second Edition: nonword repetition, blending, and elision. Comparisons are based on two-sample *t* tests or Welch's two-sample *t* tests, as appropriate, with the respective *t* values, degrees of freedom (*df*), *p* values, confidence intervals, and mean scores reported.

* *p* < .05.

In adult groups, there were differences in mean observed performance on English language measures. Monolingual adults had significantly higher mean English receptive vocabulary raw scores (*M* = 210.7) compared to Spanish–English bilinguals (*M* = 204.5), *t*(35) = 2.25, *p* = .031. Monolingual adults also had significantly higher mean blending raw scores (*M* = 28.16) compared to bilingual adults (*M* = 25.15), *t*(31) = −2.44, *p* = .021. There were no other significant differences in English language performance.

### Psychometric Function Fits

**Figure 1.**
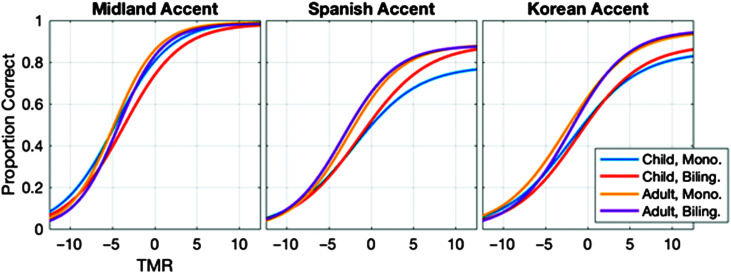
Psychometric functions fitted to group data for each accent condition and participant group. The accent condition is indicated at the top of each panel, and line colors indicate participant groups, as indicated in the legend. Mono = monolingual; Biling = bilingual; TMR = talker-to-masker ratio.

Figure 1 displays the proportion of correct responses across TMRs based on the mean performance of each language group and age group's data. The functions fit the data well, with mean *R*-squared values ranging from .97 in the Spanish accent condition (range: .75–1.00), to .98 in the Midland accent condition (range: .88–1.00), and .97 in the Korean accent condition (range: .81–1.00). From these functions, asymptotic performance, and SRT50 values were extracted for further analysis. Descriptive statistics for the upper asymptotes and SRT50s are presented in Table 2.

**Table 2.** Descriptive statistics for psychometric function parameters by group and accent condition.

Group	Accent	Parameter	*M* (*SD*)	Range
Bilingual adults	Midland	Γ	0.98 (0.01)	0.95–1.00
		SRT50	−4.20 (1.19)	−3.44–1.99
	Spanish	Γ	0.88 (0.06)	0.77–0.97
		SRT50	−2.04 (2.09)	−5.23–2.73
	Korean	Γ	0.95 (0.03)	0.88–1.00
		SRT50	−1.38 (1.38)	−3.44–1.99
Monolingual adults	Midland	Γ	0.99 (0.01)	0.96–1.00
		SRT50	−4.80 (1.13)	−6.25–1.59
	Spanish	Γ	0.88 (0.06)	0.78–1.00
		SRT50	−1.62 (1.33)	−3.63–0.80
	Korean	Γ	0.95 (0.03)	0.90–1.00
		SRT50	−2.10 (1.57)	−5.97–0.86
Bilingual children	Midland	Γ	0.98 (0.02)	0.91–1.00
		SRT50	−3.60 (1.96)	−7.77–0.47
	Spanish	Γ	0.89 (0.04)	0.82–0.97
		SRT50	−0.26 (2.06)	−4.39–3.04
	Korean	Γ	0.89 (0.06)	0.78–1.00
		SRT50	0.09 (1.99)	−4.86–3.03
Monolingual children	Midland	Γ	0.99 (0.02)	0.94–1.00
		SRT50	−4.73 (1.34)	−7.37 to −1.30
	Spanish	Γ	0.79 (0.09)	0.61–0.92
		SRT50	0.84 (2.74)	−3.71–5.18
	Korean	Γ	0.86 (0.07)	0.73–0.97
		SRT50	−0.15 (2.30)	−4.22–4.89

*Note.* Means and standard deviations are reported for upper asymptotic performance (Γ) and 50% speech recognition thresholds (SRT50) across accent conditions, separated by language group and age group.

**Figure 2.**
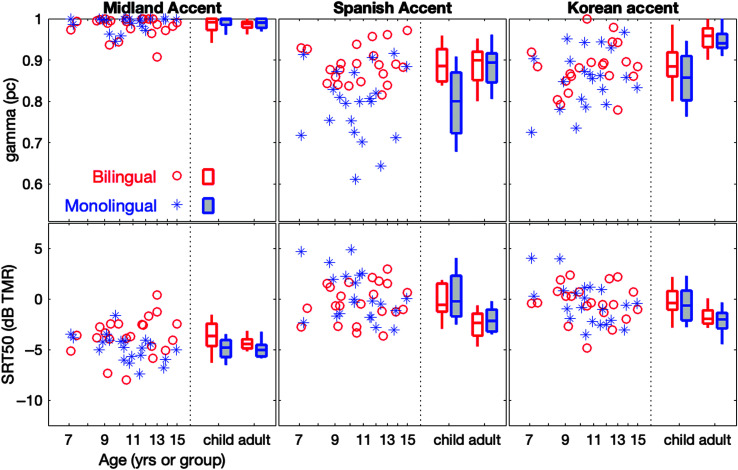
Asymptotic performance and 50% speech recognition thresholds (SRT50) for each accent condition. Asymptotic performance is shown in the top row of panels, and SRT50 is shown in the bottom row; accent condition is indicated above each column of panels. Data for individual child participants are plotted as a function of age. Symbol shape reflects language group, as defined in the legend. The distribution of data for each of the four participant groups are indicated at the right of each panel. Boxes span the 25th to 75th percentiles, horizontal lines indicate the median, and vertical lines indicate the 10th and 90th percentiles. Age group is shown on the *x*-axis, and box line color and fill indicate language group, as indicated in the legend. TMR = talker-to-masker ratio.

Figure 2 shows SRTs and upper asymptotes for individual bilingual and monolingual children, and the distribution of scores for children and adults; results are shown separately for each accent condition. In general, all four groups exhibited lower (better) thresholds in the Midland accent condition compared to the L2 accent conditions. Specifically, the increase in mean SRT50 from the Midland to the Korean accent was 2.50 dB for bilingual adults, 3.43 dB for bilingual children, 2.60 dB for monolingual adults, and 4.39 dB for monolingual children. The increase from the Midland to the Spanish accent showed similar patterns with increases of 1.79 dB for bilingual adults, 3.31 dB for bilingual children, 2.79 dB for monolingual adults, and 5.13 dB for monolingual children. Adults also had lower mean SRT50s in both L2 accent conditions compared to children. In the Spanish accent condition, adults had approximately 4.22 dB lower SRTs than children, and in the Korean accent condition they were approximately 3.91 dB lower. In contrast, the difference between child and adult groups in the Midland accent condition was 0.34 dB.

Asymptotic performance varied by participants, groups, and accent conditions. The upper asymptote, representing percent correct in quiet, was > 98% on average for the Midland English speech for all four groups. For Korean-accented speech, performance reached asymptote at a mean of 86%–95%. For all groups, Spanish-accented speech had the lowest upper asymptote, with mean values of 79%–89%, suggesting that the Spanish-accented talker was the least intelligible for the presented sentences and participants. Monolingual children performed more poorly in this condition compared to the other three groups (79% vs. 88–89%). An additional figure showing adult SRT50 and asymptotic performance as a function of participant age is available in Supplemental Material S1.

### SRTs by Accent Condition and Group

A linear mixed model assessed the effect of an interaction term that included language group (bilingual, monolingual), age group (child, adult), and accent condition (Midland, Spanish, and Korean) on SRT50s. The reference condition for this analysis was a monolingual adult in the Midland accent condition. Full results are presented in Table 3. The effects of the Korean and Spanish accent conditions were significant, showing that monolingual adults' SRTs tended to be higher in L2 accent conditions compared to the Midland accent condition. Significant interactions between both L2 accent conditions and age group were found, indicating that children had higher SRTs than adults in both the Korean and Spanish accent conditions, relative to the Midland reference. This supports the conclusion that the difference between L2-accented thresholds and Midland thresholds was larger for children than for adults, indicating greater difficulty in recognizing L2-accented speech among school-age children. There were no other significant effects to report.

**Table 3.** Linear mixed model evaluating the effect of accent condition, language group, and age group on 50% speech recognition thresholds (SRT50s).

Variable	Estimate	*SE*	*t* value	*p*
Intercept***	−4.85	0.39	−12.51	**< .001**
LanguageGroup(Bilingual)	0.62	0.55	1.13	.261
AgeGroup(Child)	0.03	0.53	0.06	.955
Accent(Korean)***	2.60	0.38	6.75	**< .001**
Accent(Spanish)***	2.79	0.38	7.27	**< .001**
LanguageGroup(Bilingual) × AgeGroup(Child)	0.55	0.75	0.73	.466
LanguageGroup(Bilingual) × Accent(Korean)	−0.09	0.54	−0.17	.862
LanguageGroup(Bilingual) × Accent(Spanish)	−1.00	0.54	−1.85	.066
**AgeGroup(Child)** × Accent(Korean)**	1.79	0.53	3.38	**.001**
**AgeGroup(Child)** × Accent(Spanish)***	2.34	0.53	4.41	**< .001**
LanguageGroup(Bilingual) × AgeGroup(Child) × Accent(Korean)	−0.86	0.75	−1.15	.250
LanguageGroup(Bilingual) × AgeGroup(Child) × Accent (Spanish)	−0.82	0.75	−1.10	.271

*Note.* The reference condition was a monolingual adult in the Midland accent condition. *SE* = standard error. Asterisks correspond to significance levels (**p < .01, ***p < .001); bolded *p* values indicate statistically significant effects (*p* < .05).

### Asymptotic Performance by Accent Condition and Group

Beta regression modeling assessed the effects of a three-way interaction term between language group, age group, and accent condition on asymptotic performance. The reference condition was a monolingual adult in the Midland accent condition. Full results are displayed in Table 4. Model results indicated that the effects of the Korean and Spanish accent conditions were significant, indicating that the reference condition (Midland) had significantly higher asymptotic performance among monolingual adults. Age group interacted with the Korean and Spanish accent conditions, indicating that children had lower asymptotic performance in the L2 accent conditions compared to adults. The three-way interaction between age group, language group, and the Spanish accent condition was significant.

**Table 4.** Beta regression model of asymptotic performance.

Variable	Estimate	*SE*	*z* value	*p*
Intercept***	3.99	0.22	18.27	**< .001**
AgeGroup(Child)	0.23	0.30	0.79	.43
Accent(Korean)***	−1.05	0.25	−4.23	**< .001**
Accent(Spanish)***	−1.90	0.23	−8.36	**< .001**
LanguageGroup(Bilingual)	−0.19	0.29	−0.63	.526
AgeGroup(Child) × Accent(Korean)***	−1.36	0.33	−4.19	**< .001**
AgeGroup(Child) × Accent(Spanish)**	−0.96	0.31	−3.12	**.002**
LanguageGroup(Bilingual) × AgeGroup(Child)	−0.13	0.41	−0.33	.741
LanguageGroup(Bilingual) × Accent(Korean)	0.31	0.34	0.90	.369
LanguageGroup(Bilingual) × Accent(Spanish)	0.14	0.31	0.44	.658
LanguageGroup(Bilingual) × AgeGroup(Child) × Accent(Korean)	0.38	0.45	0.83	.405
LanguageGroup(Bilingual) × AgeGroup(Child) × Accent(Spanish)**	0.90	0.43	2.12	**.034**

*Note.* The model included the effects of accent condition, language group, and age group, as well as a random effect of participant. For this model, the reference condition was a monolingual adult in the Midland accent condition. Asterisks correspond to significance levels (**p < .01, ***p < .001); bolded *p* values indicate statistically significant effects (*p* < .05). *SE* = standard error.

Post hoc pairwise comparisons indicated that, after adjusting for the fixed effects in the model, bilingual children had higher asymptotic performance in the Spanish accent condition than monolingual children, Estimate = 1.82, *SE* = 0.02, *t*(Inf) = 8.71, *p* < .001. There was no significant difference between child language groups in the Korean accent condition, Estimate = −37, *SE* = .18, *t*(Inf) = −1.82, *p* = .296. No other effects were significant.

### Examining the Effect of Receptive Vocabulary Size by Language Group Among Children

The recognition of L2-accented speech matures through adolescence (e.g., Bent, 2018). Given the age range of participants in the present study, we examined the effects of both chronological age and raw English receptive vocabulary scores on asymptotic performance for both bilingual and monolingual child groups. Both variables were centered prior to analysis. A beta regression model assessed the main effect of chronological age, the two-way interactions between language group and accent condition, as well as language group and English receptive vocabulary size, on asymptotic performance for child participants. The model also included a random intercept by participant. The reference level was a monolingual child in the Midland accent condition. As expected based on the previous analysis, there were effects of Korean and Spanish accents, and an interaction between language group and the Spanish accent condition. There was also a significant interaction between language groups and the Korean accent condition.

Post hoc pairwise comparisons indicated that, after adjusting for the fixed effects in the model, monolingual children had 52.3% lower odds of achieving higher asymptotic performance compared to bilinguals in the Spanish accent condition, Estimate = −.74, *SE* = 0.18, *t*(Inf) = −4.16, *p* < .001. In the Korean accent condition, monolingual children had 34.3% lower odds of achieving a higher asymptote compared to bilingual children, Estimate = −.42, *SE* = 0.19, *t*(Inf) = −2.20, *p* = .028. In terms of group differences in percentage points, bilingual children had 12 percentage-point higher asymptotic performance in the Spanish accent condition than monolingual children, and 4 percentage points higher in the Korean accent condition. No other effects were significant (full results are presented in Table 5).

**Table 5.** Beta regression model of asymptotic performance for child groups.

Variable	Estimate	*SE*	*z* value	*p*
**Intercept**	4.11	0.23	17.65	**< .001**
LanguageGroup(Bilingual)	−0.27	0.29	−0.92	.357
Accent(Korean)***	−2.33	0.24	−9.86	**< .001**
Accent(Spanish)***	−2.77	0.23	−11.81	**< .001**
Vocabulary score	0.00	0.00	0.80	.425
Age	0.01	0.05	0.17	.863
LanguageGroup(Bilingual) × Accent(Korean)**	0.69	0.31	2.24	**.025**
LanguageGroup(Bilingual) × Accent(Spanish)**	1.01	0.30	3.33	**.001**
LanguageGroup(Bilingual) × Vocabulary score	0.00	0.00	−0.23	.815

*Note.* The model included the interaction terms for accent condition and language group, language group and receptive vocabulary size, and the effect of chronological age, as well as a random effect of participant. The reference level was a monolingual child in the Midland accent condition. Asterisks correspond to significance levels (**p < .01, ***p < .001); bolded *p* values indicate statistically significant effects (*p* < .05).

## Discussion

This study evaluated how diverse linguistic experience predicts L2-accented sentence recognition by assessing SRT50s and asymptotic performance for bilingual and monolingual children and adults. It was assumed a priori that bilingual and monolingual groups differed in their experience with Spanish-accented English and Spanish phonetic features. Results indicated that there were no significant differences in SRT50s by language group among children or adults. Differences by language group emerged in asymptotic performance. As expected, Spanish–English bilingual children had higher asymptotic performance in the Spanish accent condition compared to English monolingual peers. Because bilingual children experience input from both Spanish and English at home, their linguistic environment tends to be more diverse than that of monolingual children (e.g., Levi, 2024). To examine whether this diversity supports the recognition of unfamiliar L2-accented speech, both bilingual and monolingual children completed sentence recognition testing with Korean-accented English, which was assumed to be unfamiliar to all participants. One model suggested that bilingual children outperformed monolingual children in the Korean-accented condition, though this effect was not consistently significant across models.

As expected, all four groups performed better in the L1 accent condition (Midland) compared to the L2 accent conditions (Spanish and Korean). Language groups and age groups did not significantly differ in Midland English recognition with respect to either SRT50 or asymptotic performance. This could indicate that bilingual and monolingual children were mature at recognizing Midland English sentences in noise, which is consistent with prior research (Chandni et al., 2020; Corbin et al., 2016). The stimuli used were drawn from the BKB sentence lists, which were originally developed to be used with children with typical hearing ages 5 years and older (Bench et al., 1979). It is possible that consistent performance across language groups might not hold if more linguistically complex sentence materials were used (Braza et al., 2022). Another factor contributing to the lack of group differences is that bilingual children did not differ significantly from monolingual children in terms of English vocabulary size, suggesting comparable levels of English language exposure across groups. This finding aligns with previous research, which hypothesized that a minimum level of exposure is necessary to achieve good speech-in-noise performance (Calandruccio et al., 2014; Reetzke et al., 2016). In this context, it is plausible that all participants had this requisite exposure.

### SRTs Across L2 Accent Conditions

Adults had significantly lower (better) SRT50s than children in both L2-accented conditions. Specifically, adults outperformed children by an average of 4.22 dB in the Spanish-accented condition and 3.91 dB in the Korean-accented condition. In contrast, the difference in the familiar Midland English condition was not significant (0.34 dB), suggesting that age group differences emerged in response to L2 accented speech. This finding suggests that children were not yet fully mature in L2-accented speech recognition in noise, regardless of their linguistic experience. This finding is consistent with prior research indicating the ability to recognize L2-accented speech continues to develop into late adolescence (Bent, 2018; Bent & Atagi, 2017).

No significant differences were found by language group. It is possible that asymptotic performance is more sensitive to differences in linguistic experience than SRT50. This may be because, as noise levels increase, listeners rely more on contextual cues (e.g., grammar, semantics) to facilitate speech recognition (Bent et al., 2019; Buss et al., 2019), reducing the influence of linguistic experience. In contrast, the effects of diverse linguistic input were more pronounced in quiet conditions, where listeners have greater access to phonetic detail, particularly for unfamiliar or less frequent phonetic cues.

### Asymptotic Performance Across L2 Accent Conditions

Across groups, asymptotic performance was highest in the Midland English condition, followed by Korean-accented English, with the lowest performance observed for Spanish-accented English. This likely reflects item- or talker-level variability and does not appear to interact meaningfully with the group-level effects under investigation.

For children, language group differences in asymptotic performance were observed in the Spanish accent condition. Bilingual children had 12 percentage point higher asymptotic performance compared to monolingual children, suggesting that experience with specific linguistic input generalizes to speech with similar phonetic features. These findings align with experimental research in adults (Cooper & Bradlow, 2018; Norris et al., 2003). Although no significant group differences emerged based on examination of supplemental accent data (see Supplemental Material S1), bilingual children demonstrated higher asymptotic performance. This suggests that hearing Spanish at home, along with potentially more frequent experience with Spanish-accented English, supported recognition even when participants are not able to explicitly recognize the accent from a recorded sentence. Alternative approaches to assessing accent familiarity may offer greater sensitivity and could better capture potential group differences in future research.

There were no significant differences in the ability to recognize L2-accented speech by language group for the adults, which was not predicted. In this study sample, no bilingual participants in either age group completed formal Spanish proficiency or vocabulary testing, which may be useful in future studies for fully characterizing bilingual participants' linguistic experience. Another explanation for observing a benefit of diverse language experience among children but not adult participants could be differences between children and adults in Spanish language use. The bilingual children in this study were living in Spanish-speaking households and may have more regular experience with Spanish and Spanish-accented English compared to bilingual young adults, whose linguistic environments could depend on their social networks and professional settings. A potential future direction would be to characterize language use and experience with more precision, such as through social network analysis, providing greater precision in modeling these effects. Additionally, it is possible that the semantic context in the BKB sentences reduced the effect of diverse linguistic experience on speech recognition among adults (like in McDonald et al., 2018, among children).

In the model containing only child data, bilingual children had higher asymptotic performance in Korean-accented English compared to monolingual children. This result suggests that the extent of diverse linguistic experience might generalize to unfamiliar L2-accented speech patterns, although the effect size was smaller compared to that of specific experience (4 percentage points and 12 percentage points, respectively). This contrasts with the findings of Levi (2024), which found no group differences in German-accented English word recognition between children who heard only English at home and those exposed to multiple languages. One possible explanation for this discrepancy across studies could be that Levi assessed word recognition at +5 dB SNR, whereas group differences in the present study emerged in asymptotic performance. Another key difference is that the present study assessed sentence recognition, while Levi focused on word recognition.

### Examining the Effect of Receptive Vocabulary Size by Language Group Among Children

Receptive English vocabulary and chronological age did not predict asymptotic performance in school-age children. In contrast to studies of monolingual children that have found significant effects of vocabulary size and age (e.g., Bent, 2014), our results suggest that individual differences in linguistic experience account for more variation in asymptotic performance among bilingual and monolingual children when language groups do not significantly differ in target language receptive vocabulary size. These results could be specific to bilinguals who were exposed to both languages by their fifth birthday and tend to be English dominant to some degree. They may not generalize to populations with different language dominance or language history profiles or who live in different parts of the United States, or to corpora that include less frequent or later acquired vocabulary.

## Conclusions

Among children, diverse linguistic experience associated with bilingualism predicted better perception of English produced with an L2 accent compared to that of monolingual peers. This effect was most pronounced when the prior experience matched the accent being heard. That is, experience with Spanish phonetic features and prosody was more strongly associated with recognition of Spanish-accented English than of Korean-accented English. In contrast, no such effects of diverse linguistic experience were observed in adults. These findings suggest that, in children, linguistic experience contributes to speech recognition beyond the contribution of vocabulary size. Future studies with larger samples of participants and a wider range of stimuli with different language demands are needed to strengthen these conclusions and assess their generalizability across varied language backgrounds and language dominance profiles.

## Data Availability Statement

The data sets analyzed during the current study are available from the corresponding author on reasonable request.

## Supplementary Material

10.1044/2025_JSLHR-24-00695SMS1 Supplemental Material S1 Additional information.

## Footnote

1 Two adult participants in the study were over 40 years old (one bilingual, one monolingual); the remaining adult participants ranged in age from 19 to 38 years.
