# Supplementary material for: The Effect of Diverse Linguistic Experience on Second Language–Accented English Sentence Recognition Among Monolingual and Spanish–English Bilingual Children and Adults
Source: J Speech Lang Hear Res. 2025 Dec 19;69(2):756–73. doi: 10.1044/2025_JSLHR-24-00695 (PMC12911316; doi:10.1044/2025_JSLHR-24-00695)

Supplemental Material S1. Additional information.

This schematic illustrates how the experiment was randomized between language and cognitive testing (Block 1) and speech recognition testing (Block 2) for both children and adults. All participants first completed consent and assent procedures, followed by general intake forms and language experience questionnaires, as appropriate. Participants were then randomly assigned to begin with either Block 1 (Language & Cognitive Testing) or Block 2 (Speech Recognition Testing). Within each block, task order was randomized per participant to ensure variation in administration order. After completing their assigned first block, participants proceeded to the remaining block. This procedure was identical for children and adults, with the only procedural difference being how language experience was assessed. Bilingual children’s families completed the Bilingual Input-Output Survey (BIOS; Peña, Bedore, & Rappazzo, 2003), while bilingual adults completed the Bilingual Language Profile (BLP; Birdsong et al., 2012).

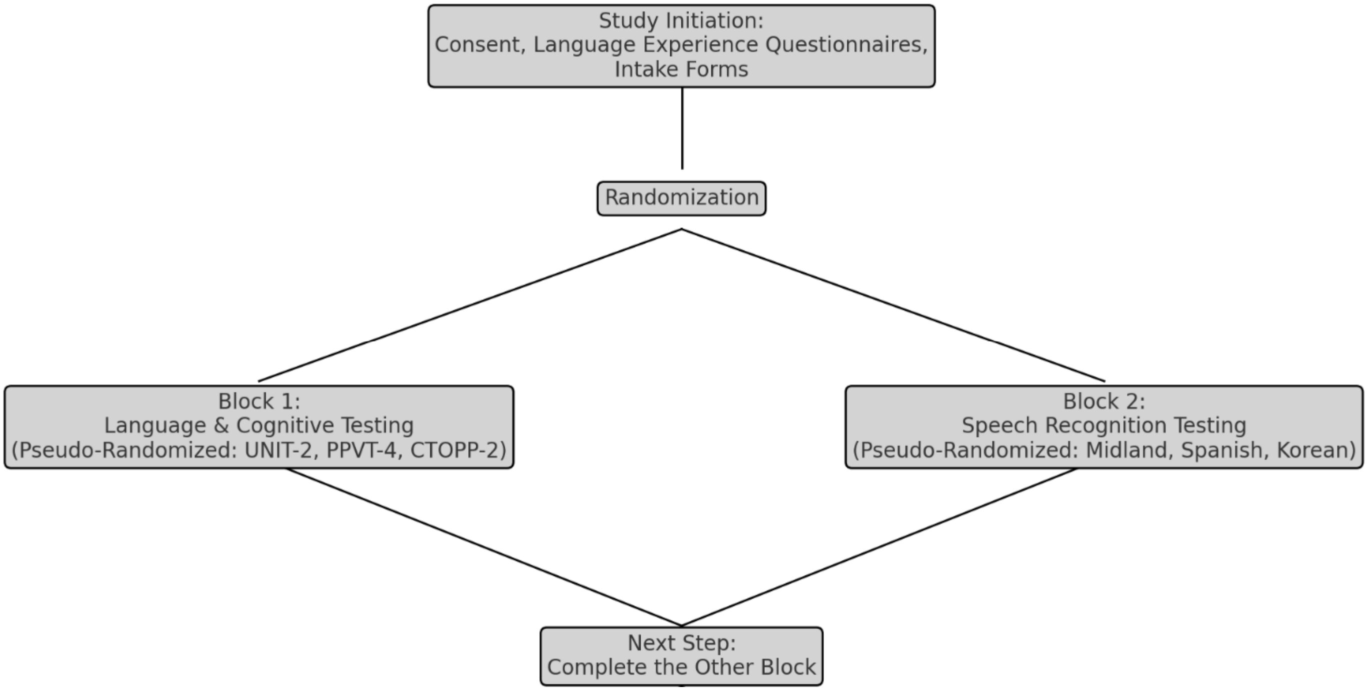

Familiarity with Korean- and Spanish-accented English was assessed at the end of speech recognition testing. The participant remained inside the booth, while the experimenter was outside using the same equipment and stimuli described for speech recognition testing.

Participants listened to two talkers per accent condition (one male, one female) from the Hoosier Database. After each sentence, participants were asked if they recognized the accent or knew anyone who spoke like the talker (i.e., "Do you know anyone who sounds like this? Have you ever heard anyone who sounds like this?"). A response of "yes" was counted as familiarity. This procedure provided a broad assessment of accent familiarity across participants and groups.

Chi-square and Fisher's exact tests assessed, as appropriate, whether bilingual and monolingual participants differed in their reported exposure to Korean- and Spanish-accented English, as well as whether familiarity varied by age (children vs. adults). For Spanish-accented English exposure, there were no significant differences between bilingual and monolingual children ($\chi^2(1) = 0.03$, $p = .86$) or between bilingual and monolingual adults ($p = 1.0$, Fisher's exact test). Similarly, for Korean-accented English exposure, neither bilingual and monolingual children ($p = .70$, Fisher's exact test) nor bilingual and monolingual adults ($p = 1.0$, Fisher's exact test) significantly differed in their reported exposure. However, significant differences emerged when comparing age groups. Adults reported significantly greater exposure to both Korean-accented English ($\chi^2(1) = 16.70$, $p < .001$), and Spanish-accented English than children ($\chi^2(1) = 15.63$, $p < .001$).

In this figure, values of SRT50 for adult participants are shown in the top row of panels, and asymptotic performance is shown in the bottom row. The accent condition is indicated above each column. Data are plotted as a function of age. Symbol shape and color correspond to language group, as reflected by the legend. Asymptotic performance is plotted as the proportion of correct responses. No age effect was predicted among adult participants, and this figure is consistent with that expectation.

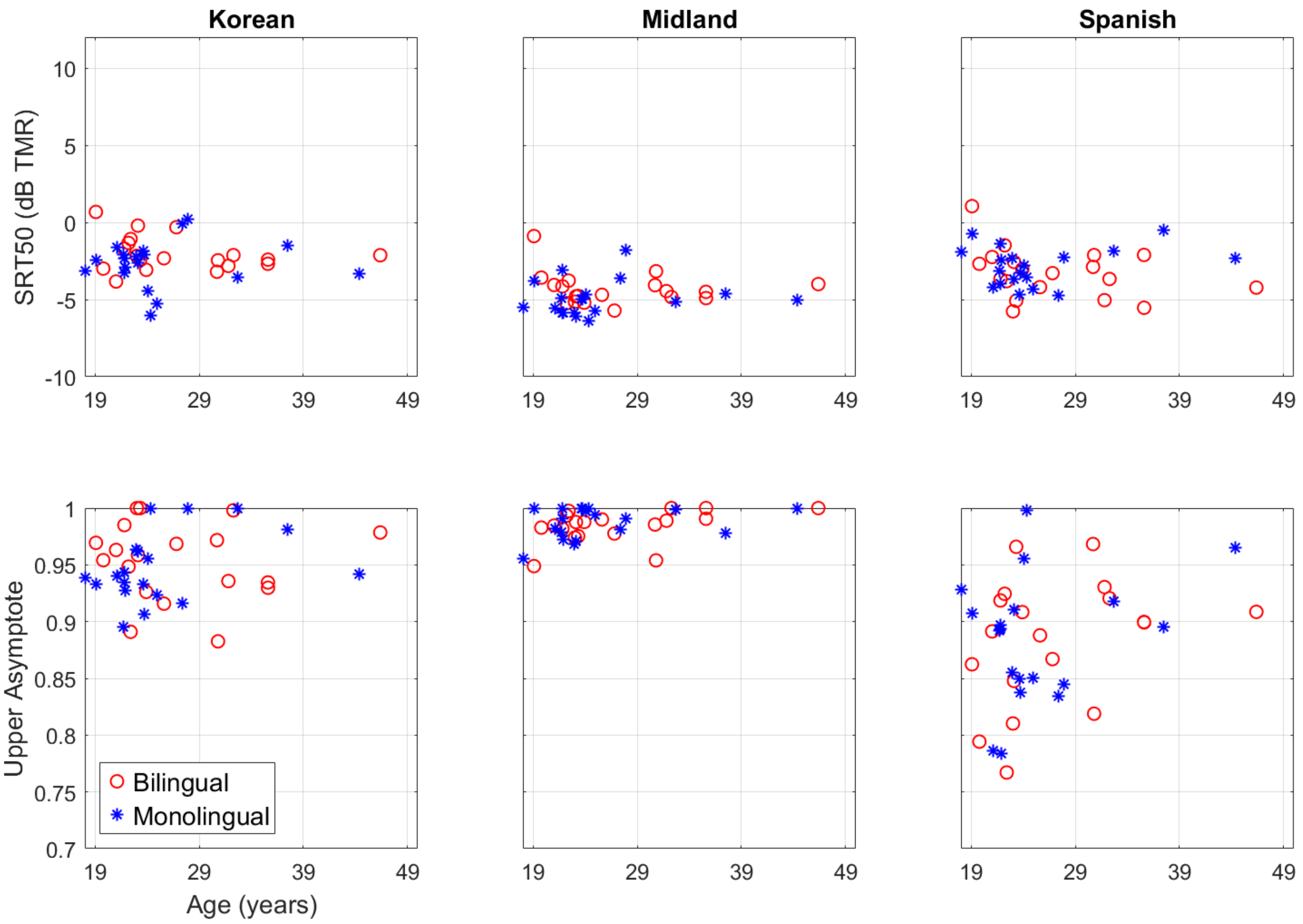

Supplement: Supplemental Material S1 [file JSLHR-69-756-s001.pdf]
